# Emerging Allergens in Goji Berry Superfruit: The Identification of New IgE Binding Proteins towards Allergic Patients’ Sera

**DOI:** 10.3390/biom10050689

**Published:** 2020-04-29

**Authors:** Carina Gabriela Uasuf, Elisabetta De Angelis, Rocco Guagnano, Rosa Pilolli, Claudia D’Anna, Danilo Villalta, Ignazio Brusca, Linda Monaci

**Affiliations:** 1 Allergy Diseases Center “Prof. Giovanni Bonsignore”, IRIB-CNR, 90146 Palermo, Italy; caren.uasuf@irib.cnr.it (C.G.U.); danna@irib.cnr.it (C.D.); 2 Institute of Sciences of Food Production, National Research Council of Italy (ISPA-CNR), 70126 Bari, Italy; elisabetta.deangelis@ispa.cnr.it (E.D.A.); rocco.guagnano@ispa.cnr.it (R.G.); rosa.pilolli@ispa.cnr.it (R.P.); 3 SSD di Immunologia e Allergologia, Ospedale S. Maria degli Angeli, 33170 Pordenone, Italy; danilo.villalta@asfo.sanita.fvg.it; 4 U.O.C. di Patologia Clinica Ospedale Buccheri La Ferla F.B.F., 90123 Palermo, Italy; brusca.ignazio@fbfpa.it

**Keywords:** goji berry, *Lycium barbarum*, food allergens, emerging allergens, proteomics, IgE binding proteins

## Abstract

The goji berry (*Lycium barbarum* L.) (GB) is gaining increasing attention with high consumption worldwide due to its exceptional nutritional value and medicinal benefits displayed in humans. Beyond their beneficial properties, GBs contain renowned allergenic proteins, and therefore deserve inclusion among the allergenic foods capable of inducing allergic reactions in sensitive consumers. GB allergy has been frequently linked to the panallergen lipid transfer protein (LTP), especially across the population of the Mediterranean area. Methods: In this study, we investigated the protein profile of GBs focusing on the most reactive proteins against immunoglobulins E (IgE) of allergic patients’ sera, as ascertained by immunoblot experiments. The protein spots displaying a clear reaction were excised, in-gel digested, and analyzed by liquid chromatography-tandem mass spectrometry (LC-MS/MS) followed by data searching against a restricted database for a reliable protein identification. Results: According to our data, three main spots were identified in GB extract as IgE binding proteins after immunoblot analysis. Some major proteins were identified and the three proteins that provided the highest reactivity were putatively attributed to vicilin and legumin proteins followed by a protein matching with 11S globulin belonging to the cupin superfamily. Finally, the whole GB protein extract was also submitted to bottom-up proteomics followed by a software-based database (DB) screening and a more exhaustive list of GB proteins was compiled.

## 1. Introduction

Goji berry (GB) which is the fruit of *Lycium barbarum* is widely consumed across the world and mostly grown in Asiatic countries but also cultivated in America, Europe, and Australia [1,2]. This fruit has been included among the officinal plants, becoming part of the traditional medicine in ancient China due to the health benefits promoted in humans. GB is a renowned source of bioactive compounds and antioxidants, as well as containing all the essential amino acids. Due to its health promoting properties, and phytochemical contents (phenolic acids, flavonoids, proanthocyanidins, iridoids, coumarins, hydrolysable tannins, carotenoids, and anthocyanins) which are endowed with antioxidant properties, this food has been included among the exotic fruits that provide beneficial properties in human beings, hence deserving the term of superfruit [3]. On the one hand, several studies have attributed various functions to this fruit such as hypoglycemic, hypolipidemic, protective towards retina cells, immunostimulatory, and anticancer which makes this fruit suitable for the diet of patients affected by different health problems [4,5]. Recently, the peculiar and healthy characteristics of this fruit has incentivated the increased consumption of GBs in Western Europe. As a direct consequence, the market has diversified the sales of this fruit commonly found on the shelves of supermarkets or in vendor machines as a single snack or incorporated in other foods. On the other hand, the increase of GB consumption has triggered, in recent years, the likelihood of displaying an allergic reaction in sensitive individuals exposed to GBs across Europe. The allergenicity of GB has not been extensively studied so far, and due to the limited information available, to date, on the containing allergenic protein sequences, more efforts are required to get deeper insights about this fruit. Some preliminary investigations [6,7,8,9] have reported the allergenic potential of this fruit capable of inducing immune reactions in sensitive individuals. GB is considered to be a new allergenic source with high prevalence of sensitization and lipid transfer protein (LTP) seems to be the major allergen involved not only in sensitization but also in cross-reactivity, according to one of the first studies accomplished by Monzón Ballarín et al. [7] which reported two cases of anaphylaxis associated with the ingestion of GB. A skin prick test with GB extract, as well as specific immunoglobulin E (IgE) to this fruit, confirmed the specific nature of the sensitization. In addition, results obtained through in vitro experiments about the allergenic profile of two patients, showed a 9 kDa band, suggesting that the corresponding protein could be attributed to LTPs. The study also demonstrated a high level of homology between LTPs from tomato and GB, which confirmed the homology existing between LTPs from different food species. Since GB belongs to the Solanaceae family it shows a high degree of homology with proteins of other plant-related foods. This accounts for the cross-reactivity phenomena with tomato and potato that belong to the same family, as documented by other authors [8,9]. In the work authored by Carnes et al., 2013 [8], the allergenic profile obtained with the serum samples collected from sensitized patients showed that more than 80% proved to react toward proteins of a lower molecular weight (6–7 kDa). Additionally, most individuals also recognized other protein bands (in the range of 26 to 65 kDa). These results suggested that sensitization to GB can be related to LTPs but also to other allergens. A recent Portuguese study using plasma of individuals with allergic history to potato and tomato proteins but without a clear history of an allergy to GB identified 11 potential allergens, of which four had never been reported in the Allergome database [10].

In the present study, we deeply investigated the other immunoreactive proteins extracted from GB and reported a proteomic investigation carried out on GB extracts tailored to the characterization of the most reactive proteins as appeared in the immunoblot experiments performed on two sera of patients sensitized to GB. Our results confirm that three bands ranging, respectively, at 48 kDa, 30 kDa, and 20–23 kDa were the most relevant for the immunoreactivity displayed and the individual spots were further submitted to high-resolution tandem mass spectrometry (HR-MS/MS) analysis and protein identification.

## 2. Materials and Methods 

### 2.1. Chemicals

Trizma base, sodium chloride, urea, ammonium bicarbonate, Tween-20, iodoacetamide (IAA), along with other chemicals for electrophoresis (dithiothreitol (DTT), sodium dodecylsulfate (SDS), glycine, glycerol, Coomassie brilliant blue-G 250, and methanol-HPLC grade) were obtained from Sigma-Aldrich (Milan, Italy). Bromophenol blue was provided by Carlo Erba Reagents (Cornaredo, Italy) and 10× Tris-tricine running buffer and 2× Tricine sample buffer were obtained from Biorad Laboratories (Segrate, Italy). Acetonitrile (Gold HPLC ultragradient) was purchased from Carlo Erba Reagents (Italy) and ultrapure water was produced by a Millipore Milli-Q system (Millipore, Bedford, MA, USA). Formic acid (MS grade) was purchased from Fluka (Milan, Italy), whereas the syringe filters (0.45 µm of porosity in regenerated cellulose (RC) and 5 µm of porosity in cellulose acetate, CA) were purchased from Sartorius (Gottingem, Germany). Trypsin (proteomic grade) for protein digestion was purchased from Promega (Milan, Italy). 

### 2.2. Clinical Cases Description 

Two Italian female patients (mean age 50 years old), Patient 1 from Palermo (South Italy) and Patient 2 from Pordenone (Northeast Italy) who reported anaphylactic symptoms after the ingestion of GB, were recruited for the study. The study was performed with the approval of the ethics committee of Policlinico—Giaccone Hospital Palermo Italy (7/2013–12.06.2013) and was in agreement with the Helsinki Declaration.

Both patients were skin prick tested with food extracts (ALK Abellò, Milan, Italy) including peach, apple, tomato, lettuce, kiwi, nuts (almond, peanut, hazelnut, cashew and chestnut), celery, milk, egg, prawn, wheat, and latex. Specific IgE to all these food extracts and molecular diagnosis (Pru p 3, Ara h 9, Cor a 8, Jug r 3, profilin, etc.) were performed (ImmunoCap, Thermo Fisher, Uppsala, Sweden) according to Brusca et al. [11]. Analysis of specific IgE to GB could not be carried out because the test was not available in our country. In addition, the skin prick test (Alk Abellò, Milan, Italy) was performed with a series of aeroallergens consisting of grasses, *Salsola kali*, *Cupressus arizonica*, *Parietaria Judaica*, Birch, Ambrosia, Mugwort, mites (*Dermatophagoides pteronyssinus* and *Dermatophagoides farinae*), epithelia (cat and dog), and mold (*Alternaria alternata* and *Aspergillus*).

Finally, both patients underwent a skin prick test with fresh GB. Serum samples from both patients were obtained after signed informed consent.

#### Skin Prick Tests

Patient 1 was a 47-year-old woman who developed grade II anaphylaxis after eating a salad with GB, lettuce, tomato, and walnut (latency <1 h), accompanied by acute rhinitis, cough, cutaneous rush, itch, difficulty swallowing, and angioedema of the uvula. She had previously been diagnosed with rhinoconjunctivitis caused by *Parietaria Judaica* and mites and had never reported food allergy symptoms. The skin prick tests with food extracts were negative, including those against profilin and LTP. IgE was measured by ImmunoCap (Thermo Fisher) with only positive values of 0.25 UI/mL to tomato, 1.46 UI/mL to almond, and 0.43 UI/mL to lettuce. In addition, the ImmunoCap ISAC (Thermo Fisher) test was performed with a positive result only to Par j 2. The skin prick tests with fresh GB (dried and wet) from the supermarket and from the restaurant where she had eaten her lunch, resulted positive (supermarket, dried GB 3 × 4 mm/wet GB 1.3 × 1.2 cm; restaurant, dried GB 5 × 4 mm/wet GB 9 × 6 mm. Histamine 4 × 4 mm/saline solution, negative).

Patient 2 was a 54-year-old woman who developed oral allergic symptoms 10 min after the ingestion of GB followed by cramps, abdominal pain, and vomiting. The symptoms disappeared within 2 h with corticosteroids and antihistaminic intravenously. The patient suffered only from celiac disease. She had asthma until the age of 6. 

The skin prick test, specific IgE to all food extracts, and ImmunoCap ISAC test (Thermo Fisher) resulted negative. The skin prick tests with the culprit GB and from another producer, resulted positive (GB 5 × 5 mm; Histamine 4 × 4 mm/saline solution, negative) 

### 2.3. Sample Preparation 

The GB samples were purchased from local retailers in semidry form and submitted to an extraction protocol optimized in the present work. Briefly, 7 g of GB were preliminarily ground in a blender (Sterilmixer 12 model 6805-50, PBI International, Milan, Italy) for 10 s (two cycles) at 17,000 rpm. Then, 5 g of the final mixture was combined with 5 mL of homogenizing buffer (Tris-HCl 0.125 mM, pH 7.4) and ground in an Ultra-turrax (Ika T25, Staufen, Germany) at 13,000 rpm for 3 min, keeping the sample in ice to prevent overheating. Finally, an aliquot of 2.5 g of slurry was combined with 5 mL of extraction buffer (Tris-HCl 0.125 mM, 50 mM NaCl, 5 M Urea, pH 7.4). After 1 min of vortex, the extract was placed in a vial and submitted to probe-based ultrasound treatment applying the following conditions: pulse on 10 s and pulse off 2 s and amplitude 70% for a total of 40 min (four cycles of 10 min each). In order to control the temperature, the vial was placed in ice to cool down the sample. Afterwards, the sample was centrifuged at 3900× *g*, the supernatant collected and filtered through a 5 µm CA syringe filter. Then, the samples were stored at −20 °C until use. The experiment was performed in duplicate.

### 2.4. Electrophoretic Separation and Protein Assay

The protein content of GB extracts was measured using Lowry assay (DC protein Assay, Bio-Rad Laboratories). Bovine serum albumin (BSA) was used as the standard protein and a calibration curve within the range of 0.125–1 mg/mL was built up. The GB protein content was calculated as mg/albumin equivalent. The absorbance was measured at 750 nm wavelength.

The protein fraction of GB was firstly separated by sodium dodecylsulfate-polyacrylamide gel electrophoresis (SDS-PAGE) on an AnykD^TM^ TGX polyacrylamide pre-cast gels (8.6 cm × 6.7 cm × 1 mm, Bio-Rad Laboratories. Presumably corresponding to 8–16% polyacrylamide composition). Before gel analysis, a calculated amount of sample (corresponding to 105 µg of proteins) was mixed (1:1 ratio) with a Laemmli buffer (62.5 mM Tris-HCl, pH 6.8, 25% glycerol, 2% SDS, 0.01% Bromophenol Blue, 100 mM DTT), and then kept at 100 °C for 5 min to allow denaturation. PAGE gels were run in the Mini-Protean Tetra Cell equipment (Bio-Rad Laboratories) with TGS (25 mM Tris, 192 mM Glycine, 0.1% SDS) running buffer at the following conditions: 60 V for 15 min and 90 V until the end of the run. Gels were stained using a solution of Coomassie Brilliant Blue G-250, and the bands were detected on a ChemiDoc™ Imaging System (Bio-Rad Laboratories). As a protein molecular weight reference, Precision Plus Protein™ All Blue Standards (10–250 kDa, Bio-Rad Laboratories) was used. 

In order to have more information about low molecular weight proteins that are, in general, scarcely resolved by conventional Tris-glycine SDS-PAGE, goji proteins were additionally separated by Tris-tricine PAGE approach that deeply increased the resolution of proteins banding in the range of 2–37 kDa. A 16.5% precast gel (8.6 cm × 6.7 cm × 1 mm, Bio-Rad Laboratories) was used for this purpose and 120 µg of goji proteins were loaded into each lane. Before loading, samples were mixed (ratio 1:1) with tricine sample buffer (200 mM Tris-HCl, pH 6.8, 2% SDS, 40% glycerol, 0.04% Coomassie Brilliant Blue G-250, 125 mM DTT) and denatured at 100 °C for 5 min. Gels were run in the Mini-Protean Tetra Cell equipment (Bio-Rad Laboratories) with Tris-tricine (0.1 M Tris, 0.1 M Tricine, 0.1% SDS, pH 8.3) running buffer at the following conditions: 60 V for 15 min and 100 V until the end of the run. Precision Plus Protein Dual Xtra Standard (Bio-Rad Laboratories) was used as a protein ladder. 

### 2.5. Proteomic Investigation

#### 2.5.1. Protein Profiling of Goji Berries

The protein identification of GB was obtained following the bottom-up proteomic approach where proteins are identified starting from the peptide mixture arisen from the digestion operated by selected proteolytic enzyme, such as trypsin. Briefly, GB extract (*n* = 2) was diluted 10 times with 55.5 mM ammonium bicarbonate (AB) buffer and an aliquot of 250 µL denatured at 95 °C for 15 min. Subsequently, 12.5 µL of a 50 mM dithiothreitol (DTT) solution were added for protein reduction followed by 30 min incubation in a thermoshaker at 60 °C. After cooling the sample at room temperature, 25 µL of a IAA solution 100 mM were added and the mixture was placed in the dark for 30 min at room temperature. Finally, 12 µL of a trypsin solution (1 µg/µL in acetic acid 50 mM) were added to the sample so that a ratio of 1:50 (enzyme/protein) was attained to facilitate a complete enzymatic digestion. Reaction was halted after 14 h by acidifying the sample with 5 µL of HCl 3 M, then, the final extract was centrifuged at 9500× *g* for 10 min and filtered on 0.45 µm RC filters before injection. The experiment was accomplished in duplicate and each sample was injected twice in the liquid chromatography-mass spectrometry (LC-MS) instrument.

#### 2.5.2. In-Gel Protein Digestion

The most informative protein bands of GB Tris-glycine and Tris-tricine electrophoretic profile were cut from the polyacrylamide gel and submitted to in-gel digestion procedure according to the protocol reported in our previous work [12]. Finally, each sample was resuspended in 50 µl of H_2_O/ACN, 90:10 + 0.1% formic acid (*v*/*v*) and 20 µl were further injected into LC-MS apparatus.

#### 2.5.3. LC-HR-MS/MS Analysis and Bioinformatics

Protein bands and peptide mixture arisen from digestion of goji protein extract were analyzed by liquid chromatography tandem mass spectrometry (LC-MS/MS) system followed by a bioinformatic search for identification purpose. Specifically, a Q-Exactive™ Plus Hybrid Quadrupole-Orbitrap™ Mass Spectrometer coupled to a Ultra-High-Performance Liquid Chromatography (UHPLC) pump system (Thermo Fisher Scientific, San José, CA, USA) was used. LC separation was accomplished on a reversed phase Aeris peptide analytical column (internal diameter 2.1 mm, length 150 mm, particle size 3.6 μm, porosity 100 Å, Phenomenex, Torrance, CA, USA) at a flow rate of 200 μl/mL according to the following gradient elution ((A) aqueous formic acid 0.1% solution (*v*/*v*) and (B) 0.1% (*v*/*v*) formic acid in acetonitrile): solvent B was set at 5% for 1 min, then delivered by a linear gradient from 5 to 55% in 60 min and kept constant for 5 min, and then increased to 90% in 1 min and maintained at these conditions for 10 min. Solvent B was delivered to 5% in 1 min before column re-equilibration (15 min). The column temperature was kept constant at 25 °C during the run and the sample volume injected was 20 µl. MS Spectra were acquired in the mass range 200–2000 m/z in a positive ion mode by running the instrument in a data-dependent MS/MS acquisition mode (dd-MS2). Up to 10 most intense ions in MS1 were selected for subsequent fragmentation in MS/MS mode. A resolving power of 70,000 full width at half maximum (FWHM), a microscan of 1, an automatic gain control (AGC) target of 1e6, and a maximum injection time (IT) of 30 ms were set to generate the precursor spectra (full MS analysis). The parameters for MS2 fragmentation experiments were set as the following: resolving power 17,500 FWHM, microscan of 1, AGC target 1e5, maximum IT 60 ms, loop count 10, MSX count 1, isolation window of 2 m/z, isolation offset 0.4 m/z, and stepped normalized collision energy (NCE) at 25–27–30 eV; as for dd-setting, maximum AGC target was set at 5.10e1 and 3.00e2 for bands and total goji proteins analysis, respectively. Finally, dynamic exclusion was set at 20 s, peptide match set to preferred, and exclude isotopes enabled. All ions with a charge higher than 4 were excluded. The electrospray ionization (ESI) source conditions were as follows: spray voltage at 3.4 kV, capillary temperature at 320 °C, sheath gas and auxiliary gas flow rates at 25 and 15 arbitrary units, respectively, S-lens at 55.

The bioinformatic search was accomplished by using the commercial software Proteome Discoverer™ version 2.1 (Thermo-Fisher-Scientific, San Josè, CA, USA). After processing the MS raw spectra, protein identification was achieved by Sequest HT search against *Eudicotyledons* (taxonomy ID 71240) database extracted by UniProt DB. The identification of tryptic peptides which were originated by digestion experiments was accomplished by setting at 5 ppm and 0.05 Da, respectively, the mass tolerance on the precursor and fragment ions [13]. After validation of the peptide spectrum matches by visual inspection of the analyst, only proteins identified by a minimum of two peptides detected with a high/medium confidence (false discovery rate, FDR <1% and <5%, respectively) were taken into account.

### 2.6. Immunoblotting Experiment

Tris-glycine and Tris-tricine PAGE of the goji sample (corresponding to 105 and 120 µg of proteins loaded, respectively) were electroblotted onto a 0.2 µm Polyvinylidene Difluoride (PVDF) membrane (Bio-Rad Laboratories) using a Trans-Blot Cell from BioRad (Bio-Rad Laboratories) for 7 min (1.3 A, 25 V). Immunoblotting experiments were accomplished according to the protocol described by Bavaro et al., 2019 [14]. As the primary antibody, individual sera with different dilution factors and a pooled sera of 2 patients allergic to goji and previously diluted in TBS-T at the ratio 1:20 were used, while as a secondary antibody a Goat Anti-Rabbit IgG (H + L) HRP Conjugate (Bio-Rad Laboratories) previously diluted 1/5000 (*v/v*) in TBS-T was added. Final images were acquired on a ChemiDoc^TM^ MP Imaging System (Bio-Rad Laboratories). For the negative control, sera of non-atopic patients were also analyzed by immunoblotting and the absence of any reactivity of the negative control was first assessed.

## 3. Results

### 3.1. Gel Electrophoresis Separation and Immunoblot Analysis 

**Figure 1:**
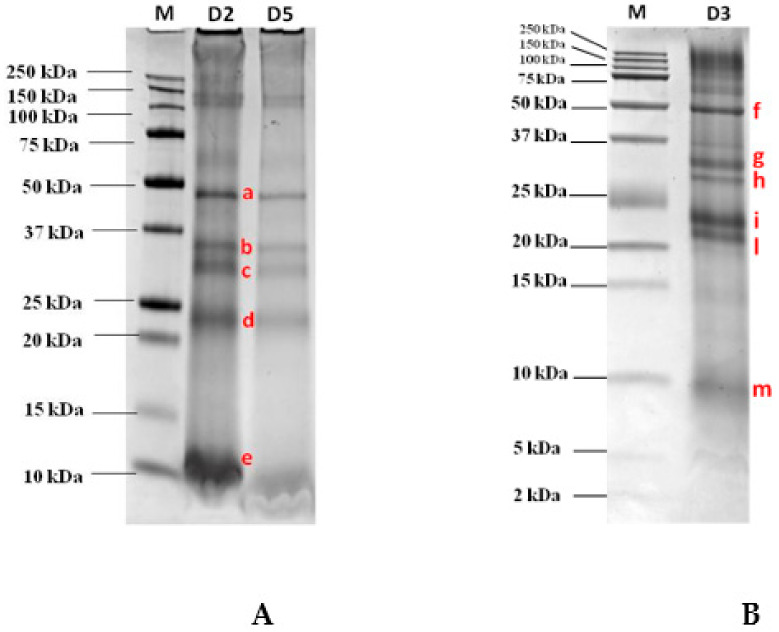
Separation on AnykD^TM^ gel (Tris-glycine PAGE) and Tris-tricine 16.5% gel (Tris-tricine PAGE) of a goji berry (GB) protein extract loaded at several dilutions. (**A**) 1:2 (D2) corresponding to 105 µg of proteins, 1:5 (D5) corresponding to 60 µg of proteins; (**B**) 1:3 (D3) corresponding to 120 µg of proteins) after staining with Comassie blue. Bands submitted to in-gel tryptic digestion for further mass spectrometric analysis, were marked with letters (a–e, (**A**) and f–m, (**B**)).

The optimized extraction protocol as described in the Material and Methods Section, was applied to semidry GB aiming at maximizing the amount of final proteins extracted. After protein quantification, the whole GB extract was loaded on the SDS gel for protein separation in order to have the final protein pattern under reduced conditions. As pictured in Figure 1A, the pattern clearly shows the four most intense protein bands migrating in three main areas of the gel and covering the molecular weight (MW) ranges 48 kDa, 35–30 kDa, and 23 kDa on AnykD^TM^ gel (8–16% polyacrilamide). In addition, a protein displaying a high migration, although if not well defined, was also displayed at the bottom of the gel close to the 10 kDa band. To better explore the lower MW range below 25 kDa and in order to confirm their detectability, a second electrophoretic separation was accomplished on a Tris-tricine gel at 16.5% of acrylamide which was capable of better resolving the proteins in the lower MW range. Figure 1B shows the separation accomplished on that specific gel (16.5% poliacrylamide) by loading an amount of GB proteins as low as 120 µg. Although this further separation confirms the presence of certain proteins at the higher MW that were already clearly visualized in the 8–16% gel, in the medium and lower MW ranges the proteins appeared to be better resolved. As reported in the figure, two bands were detected in the range 30–35 kDa in line with that shown in Figure 1A with respect to the AnykD^TM^ gel, while the unique band that was detected in the previous SDS gel at the MW of approximately 23 kDa in this gel appeared to be split in two resolved proteins migrating, respectively, at 21 and 23 kDa. In the bottom part of the gel, another faint protein banding at 9 kDa was also clearly detected and putatively assigned to the 9 kDa LTP. This protein represents a renowned reactive protein found in several fruits (i.e., peach, cherry, apricot, and apple) as widely reported by other authors [15,16,17,18].

### 3.2. Immunoblot Analysis After Electrophoretic Separation 

**Figure 2:**
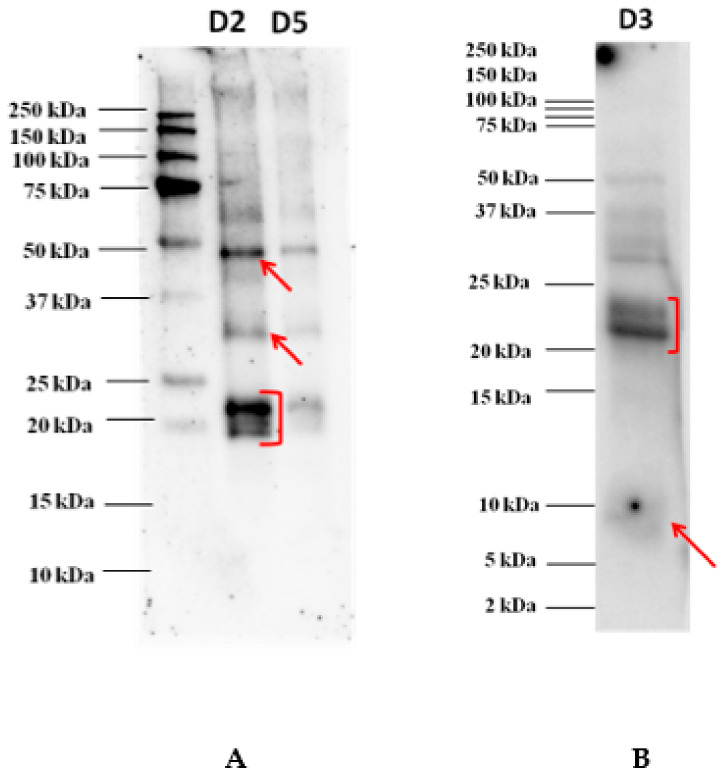
Immunoblot analysis after goji protein separation on AnykD^TM^ gel (**A**) and on Tris-tricine gel (at dilution D3) (**B**). Different dilutions of goji extract were loaded, namely 1:2 (D2, corresponding to 105 µg of proteins) and 1:5 (D5, corresponding to 60 µg of proteins) for AnykD^TM^ gel and 1:3 (D3, corresponding to 120 µg of proteins) for Tris-tricine gel. Serum was diluted 1:20 in TBS-T before incubation.

The proteins separated onto the Tris-glycine electrophoresis gel (AnykD^TM^) were electroblotted on a 0.2 µm PVDF membrane before incubation with each individual serum of allergic patients. The image acquired of an immunoblot experiment developed after incubation with the serum of one allergic patient is shown in Figure 2. According to our findings, reactivity profiles appeared to be very similar with the most reactive proteins focused in the area 48 kDa, a faint spot around 30 kDa, and another clear spot in the area 20–23 kDa where three overlapping bands were highlighted (see Figure 2). No band reacted against IgE in the lower MW. In order to investigate on the reactivity of proteins banding in the lower MW range, Western blot experiments were performed with the pooled sera after protein separation on Tris-tricine gel, as reported in Figure 1B. The results showed a rather faint reactive band detected below 10 kDa, putatively attributed to 9 kDa LTP protein, which was not displayed in the previous immunoblot where proteins were separated on an AnykD^TM^ gel. This highlights the importance of selecting the most appropriate gel for a better visualization and resolution of the proteins falling in the lower MW range. Tris-tricine gel was confirmed to be the preferred approach for resolving proteins smaller than 30 kDa and for the isolation of extremely hydrophobic proteins prior to proteomic analysis. In this case, such separation proved to be suitable in terms of best resolution of protein bands below 25 kDa.

As appearing from the figure, the two bands detected in the range 21–23 kDa also showed to react at a lower extent with IgE of the pooled serum, thus, confirming what was highlighted in the previous immunoblot. All the proteins detected that proved to react against allergic patients’ sera were cut from the corresponding gel and subsequently prepared for the LC-HRMS/MS analysis for final protein identification. 

### 3.3. LC-HR-MS/MS Identification of the Excised Protein Bands

The most intense bands detected in the AnykD^TM^ gel and marked with the letters a, b, c, d, e (see Figure 1A) and the bands labelled as f, g, h, i, l, m visualized in the Tris-tricine gel (see Figure 1B) were excised, decolored, and submitted to in-gel tryptic digestion before LC-HR-MS/MS analysis. All data collected were further processed using the commercial software Proteome Discoverer that enabled a straightforward identification of the candidate proteins by interrogating huge protein databases available online (UniProt). In Table 1 are reported the proteins identified for each selected spot (a–e bands excised from the gel AnykD^TM^ and f–m bands from the Tris-tricine gel) analyzed by MS/MS (see Figure 1A) along with the list of unique peptides attributed to each putative protein by setting at least medium confidence level in sequence identification (FDR <5%). The database (DB) screened was *Eudicotyledons*, namely a restriction of *Viridiplantae* DB. According to the results, on the a band (Figure 1A) at approximately 48 kDa, two separate proteins were identified, specifically a vicilin and a vicilin-like protein with four unique peptides detected in both cases. In the lower MW comprised between 30 and 35 kDa two different bands, b and c (Figure 1A), were displayed and attributed to fibrillin and to an uncharacterized protein from *Solanum lycopersicum* after proteomic analysis. This uncharacterized protein was a cupin-like protein and as such, endowed with allergenic potential. Conversely, the spot banding in the range 22–24 kDa named “d” (Figure 1A) was attributed to two different proteins coeluting in the same spot, namely glutelin and legumin A, this last legumin representing a renowned allergic 11S protein characterizing nuts and in particular found in peanut, hazelnut, cashew, walnut, and sesame seeds [19,20] and responsible for inducing allergic reactions in sensitive individuals. Similar results were obtained by analyzing the bands f–m (Figure 1B) excised from Tris-tricine gel, each corresponding to different spot in the AnykD^TM^ protein profile.

### 3.4. Bottom-Up Proteomics of Goji Berry Extract 

High throughput identification of peptides from tandem mass spectrometry data is widely used for protein identification in modern proteomics. A key step in mass spectrometry is to identify the peptide sequence that, most probably, gives rise to each observed spectrum. This is often tackled using a large or customized database search, i.e., each observed spectrum is compared against a large number of theoretical “expected” spectra predicted from candidate peptide sequences in a database. Common approaches leading to protein identification results are based on likelihood-based, scoring criterion, which are constructed from the set of best scores produced by large collections of MS/MS spectra using searching engines such as Sequest HT. With the aim to overcome limitations posed by the low levels of proteins of the spots detected in the gel that could impact reliable protein identification by MS/MS analysis and in order to provide an overall picture of proteins contained in goji fruit, the whole protein extract described in the Materials and Methods Section was submitted to the entire proteomic workflow including tryptic digestion and LC-HR-MS/MS analysis. All data collected were processed via Proteome Discoverer software using the searching algorithm Sequest HT and specific filters were set to accrue the confidence in protein identification. Table 2 reports a list of the first 30 hits retrieved after the database screening (filtered on a total of 115 hits, the complete list is reported in Table S1 of Supplementary Materials) along with the number of (unique) peptides detected for each putative protein, the protein coverage, and the Sequest HT score (indicative value of the reliability of the identification, specifically it is calculated by summing all peptide Xcorr values above the specified score threshold). As shown in the table, several proteins belonging to different species were identified in the whole GB extract after searching peptide sequences against the *Eudicotyledons* database and, in particular, the following deserve major attention due to their allergenic potential: glutelin and legumin with a total of 12 peptides each were the proteins with the highest coverage followed by vicilin, cupin proteins, and non-specific lipid transfer proteins. In particular, these last proteins are also listed in the allergome DB (www.allergome.org), which is a collection of allergenic proteins bearing specific epitopic sequences, and therefore considered to be capable of triggering allergic reactions in sensitive individuals. By comparing these data arisen from bottom-up proteomics of the whole GB extract with the identification carried out by analyzing the individual spots excised from the SDS gel, it is possible to confirm the identity of proteins that were already identified by in-gel digestion and LC-HR-MS/MS analysis of the resulting peptide mixture. These data give rise to draw a final list of most reliable allergenic proteins identified in GB extract such as vicilin, legumin, LTP, and cupin proteins. Then, a further investigation was accomplished to look for cross-reactivity shown by the identified proteins against other proteins present in the database based on the % of homology shared. The results of this search by screening the sequence in the allergenonline platform (www.allergenonline.org) (based on full length alignment) are summarized in Table S2 of the Supplementary Materials. As depicted, the higher the percentage of identity, the higher the likelihood of cross-reactivity among other species as in the case of 55% homology reported between the uncharacterized protein (accession A0A3Q7IWI5) and the 11*S* globulin (*Bertholletia excelsa*). Other proteins scoring lower in Table S2 displayed even higher homologies, as is the case of high identity found between other detected proteins and enolases or LTPs.

## 4. Discussion

GB is considered a high value fruit due to its significant content of health promoting compounds, namely polysaccharides (displaying important biological activities) and phytochemicals (phenolic acids, flavonoids, proanthocyanidins, iridoids, coumarins, hydrolysable tannins, carotenoids, and anthocyanins) endowed with antioxidant properties [3] which are beneficial for humans’ health. Due to the wide distribution of this fruit across the world, its consumption has hugely increased over the last years. Additionally, the reactivity toward this fruit has emerged among the general population, with reports of severe cases of allergy to GB being described in several countries. One of the first cases of severe reaction to GB was documented by Monzón Ballarín et al., 2011 [7]. The authors described two cases of patients reporting allergic symptoms after GB ingestion, one of which was under anaphylactic reaction. A protein banding at 9 kDa, attributed to LTP, was revealed to be the most reactive by immunoblot experiments with sera of allergic patients [7]. In another study [6], using skip prick testing, it was reported that of 30 patients allergic to plant foods, approximately 77% reacted towards GB. In addition, positivity to GB was associated with positivity to peach peel and to the panallergen non-specific LTP. The same authors found that 7 kDa was the protein most reactive by immunoblot experiments carried out with most of sera and was putatively attributed to LTPs, as retrieved by inhibition experiments using purified LTP from peach. This was followed by a second reactive protein banding at 50 kDa MW that was not identified and was considered to play an important role in sensitization to GB [6]. The same findings were confirmed by Carnes et al., 2013, who screened a total of 566 individuals with respiratory or cutaneous symptoms [8]. By skin prick test with GB extract, approximately 6% were found positive, while 94% were sensitized to other allergens; confirming that the immunogenicity found with 7 kDa band was attributed to LTP. The same authors also found cross-reactivity with tomato, tobacco, nut mix, Artemisia pollen, and purified Lyc e 3 and Pru p 3 [8]. However, due to the lack of studies accomplished in this regard there is a paucity of information available about the extent of allergy to GB across the European population and about allergenic proteins identified in GB fruit. In this investigation, we report a study aimed at characterizing the most reactive proteins identified in GB extracts that proved to bind IgE of allergic individuals as highlighted by the immunoblot experiments carried out on sera of two patients that displayed clear anaphylactic symptoms upon GB ingestion. According to the results obtained after LC-HR-MS/MS followed by database searching, two main proteins were identified as capable of binding IgE of allergic patients namely vicilin-like protein and legumin, whereas a weaker spot was displayed in correspondence of LTP. Legumin and vicilin-like proteins are storage proteins typically found in legume seeds. They belong to the cupin superfamily forming the two major classes of globulins, namely vicilins (7S globulins) and legumins (12S globulins); in particular, based on their sedimentation coefficients, they can be graded into 11S legumin type and 7S vicilin type [21]. Globulins are known to define the nutritional quality of seeds; however, they are also involved in sucrose binding, desiccation, defense against microbes, hormone binding, oxidative stress, etc. The major drawback with globulins is their tendency to bind to IgE [21], and therefore were included in the list of proteins endowed with allergenic potential. Non-specific lipid transfer protein is by far the most frequent cause of primary food allergy in adults living in the Mediterranean area and it is also the main cause triggering food-dependent anaphylactic reactions. LTPs belong to the family of pathogenesis-related (PR) proteins and represent panallergens common to phylogenetically close foods. The most important aspects of LTP sensitization and allergy, along with the issues regarding the cross-reactivity between LTPs present in botanically related and unrelated foods have been reviewed by Asero et al. [22]. In GB, this corresponds to the Lyc ba 3 protein annoverated in Allergome DB. LTP is located in the superficial layers in a number of fruits, including *Rosaceae* (peach, apple, pear, plum, cherry, and apricot), melon, and watermelon, having a defensive role in the plant. The plant LTP family itself includes the following two subfamilies showing a slightly different molecular mass: 9 kDa LTP 1, encompassing the large majority of these proteins, and the 7 kDa LTP 2, including a much more limited number of representatives, mostly in cereals and tomato. The 7 kDa LTP in tomato seeds shares some structural features with peach LTP but displays individual features responsible for monospecific IgE binding. Although the two families share the general molecular structure, they have a low sequence similarity (about 30% identity) and differ in cysteine residues sited along the molecules. They are rich in disulphur bridges and are found highly resistant to heat and to intestinal proteolysis, besides showing a good stability to pH changes. In light of this, LTP could be considered a good candidate as a primary sensitizer, although in this case a weak IgE-reactivity mediated by LTP in the tested allergic consumers was observed. In conclusion, our results showed that the major reactivity displayed towards IgE of allergic patients’ sera collected in two opposite parts of Italy was mainly attributed to two classes of proteins including legumin-like and vicilin protein followed by 11S globulin belonging to cupins. In contrast, despite what has been reported by other authors on sera of different geographic origin, LTP protein deserves a lower role in this investigation and did not prove to significantly react towards IgE of the selected allergic subjects enrolled in this study. As expected, the molecular allergology pattern of food allergy can vary from country to country and also within the same country depending on intrinsic factors of the population. As a result, it is reasonable to find a different immunoreactive pattern for GB although other authors have reported that it strictly depending on the place of origin of the collected sera [23,24,25]. This should encourage conducting a wider investigation on consumers allergic to GB across the population to have more insights into the reactive proteins capable of representing a menace for sensitive consumers and on the cross-reactivity that can occur with other plant-related foods.

## 5. Conclusions

The results of our proteomic investigation on GB extracts which aimed to characterize the most reactive proteins towards sera of allergic patients confirmed that three bands banding, respectively, at 48 kDa, 30 kDa, and in the range 20–23 kDa were the most relevant for the allergenic potential. The LC-HR-MS/MS analysis attributed these most reactive spots to vicilin and legumin proteins and to 11*S* globulin belonging to the cupin superfamily, as assessed by immunoblot experiments.

## Figures and Tables

**Table 1:** Protein identification by LC-HR-MS/MS analysis of excised protein spots from AnykD^TM^ (band a–e) and Tris-tricine gel (bands f–m). In bold are reported the putatively proteins reacting to IgE of allergic sera.

AnyKD Gel	Tris-Tricine Gel	Putative Identification (Accession)	Type of Protein	Coverage (%)	Peptides (Unique)	MW	Protein Score
a	F	A0A2R2JFS1	Vicilin, OS = *Solanum melongena*	14.08	6 (4)	48.6	3.17
		A0A1S4CJA8	Vicilin-like, OS = *Nicotiana tabacum*	6.53	7 (4)	94.6	2.7
b	G	A0A0A7DVY6	Fibrillin, OS = *Lycium barbarum*	51.09	13 (13)	35.3	16.74
**c**	H	A0A3Q7IWI5	Uncharacterized protein, OS = *Solanum lycopersicum*	12.87	6 (6)	53.3	3.75
		A0A0A7DVY6 (Fibrillin)	Fibrillin, OS = *Lycium barbarum*	29.28	8 (8)	35.3	1.8
d	I	A0A1J6JXR5 (glutelin)	Glutelin-like, OS = Nicotiana attenuata	14.73	9 (4)	53.6	10.93
	L	A0A2G2ZXN5 (legumin)	Legumin-like, OS = Capsicum annuum	9.72	6 (1)	52.9	3.82
e	M	B3A0N2	LTP, OS = Lycium barbarum (fragment)	39.21	1 (1)	4.9	6.52

MW: molecular weight; OS: prganism species

**Table 2:** Protein identification in protein extract of GB (goji berry) by bottom-up proteomics by filtering hits to the first 30 proteins identified at the highest score.

N.	Accession	Description	Coverage (%)	Peptides (Unique)	PSMs	Score Sequest HT
1	A0A0A7DVY6	Fibrillin OS = *Lycium barbarum*	69.16	18 (13)	302	524.75
2	A0A3Q7IWI5	Uncharacterized protein OS = *Solanum lycopersicum*	23.21	11 (4)	187	271.84
3	A0A1J6JXR5	Glutelin type-b 5 OS = *Nicotiana attenuata*	22.53	12 (4)	196	247.78
4	A0A1U7X9B2	11*S* globulin seed storage protein 2-like OS = *Nicotiana sylvestris*	19.02	10 (2)	116	87.44
5	M1B1M5	Uncharacterized protein OS = *Solanum tuberosum*	15.81	9 (1)	115	81.78
6	A0A3Q7FEY7	Uncharacterized protein OS = *Solanum lycopersicum*	4.52	8 (1)	81	67.30
7	A0A1J6L9S7	11s globulin subunit beta OS = *Nicotiana attenuata*	17.10	13 (1)	124	66.05
8	A0A1U7VG63	Legumin B-like OS = *Nicotiana sylvestris*	21.17	12 (2)	119	55.54
9	A0A2G9GMS2	Uncharacterized protein OS = *Handroanthus impetiginosus*	12.66	4 (1)	41	53.98
10	A0A144YUS5	Ribulose bisphosphate carboxylase large chain OS = *Iochroma lehmannii*	23.69	10 (2)	75	53.58
11	A0A022S2J1	Uncharacterized protein OS = *Erythranthe guttata*	5.18	3 (2)	48	53.58
12	G0WZI6	Ribulose bisphosphate carboxylase large chain (Fragment) OS = *Solanum chenopodioides*	16.56	7 (1)	42	51.71
13	A0A2G2X5V4	11*S* globulin seed storage protein 2 OS = *Capsicum baccatum*	14.95	9 (2)	89	50.63
14	A0A1U7W1Q9	Vicilin-like antimicrobial peptides 2-3 OS = *Nicotiana sylvestris*	10.80	9 (6)	92	49.82
15	M1A8H0	Uncharacterized protein OS = *Solanum tuberosum*	66.67	5 (4)	56	46.28
16	A0A2R2JFS1	SM80.1 Vicilin OS = *Solanum melongena*	14.79	7 (3)	77	44.34
17	Q948T8	Histone H4 (Fragment) OS=Citrus jambhiri	45.10	5 (2)	40	43.92
18	A0A1U8GRB4	catechol oxidase B, chloroplastic OS = *Capsicum annuum*	19.35	10 (2)	74	41.66
19	A0A2C9V2W2	Uncharacterized protein OS = *Manihot esculenta*	22.99	10 (0)	91	38.72
20	A0A103XB50	Histone H4 OS = *Cynara cardunculus* var. scolymus	45.63	5 (1)	46	36.74
21	M1ANI2	Uncharacterized protein OS = *Solanum tuberosum*	49.59	6 (3)	37	35.68
22	A0A1U8F773	Uncharacterized protein OS = *Capsicum annuum*	8.89	4 (1)	47	34.82
23	A0A059AJT0	Uncharacterized protein OS = *Eucalyptus grandis*	18.40	7 (1)	75	34.60
24	A0A1S4BK33	11S globulin subunit beta-like OS = *Nicotiana tabacum*	11.58	7 (2)	70	33.12
25	A7UGG9	Non-specific lipid-transfer protein OS = *Solanum tuberosum*	17.54	2 (1)	21	30.61
26	Q40151	Hsc70 OS = *Solanum lycopersicum*	18.28	6 (1)	63	27.21
27	B3A0N2	Non-specific lipid-transfer protein (Fragments) OS *= Lycium barbarum*	39.22	1 (1)	21	26.93
28	F1DBB8	Chloroplast polyphenol oxidase OS = *Solanum melongena*	20.31	7 (1)	68	26.24
29	C0SQK3	Elongation factor1-alpha (Fragment) OS = *Rosa hybrid cultivar*	14.63	3 (0)	35	26.01
30	K4D1U9	Non-specific lipid-transfer protein OS= *Solanum lycopersicum*	25.44	2 (1)	27	25.73

OS: organism species; PSMs: peptide-spectrum-matches.

## Supplementary Materials

The following are available online at https://www.mdpi.com/2218-273X/10/5/689/s1, Table S1: Full list of the total protein hits retrieved by Proetome discoverer software screening; Table S2: Cross-reactivity of the protein identified in the total GB digest by proteomic discovery MS analysis and by searching and aligning the full length sequences on www.allergenonline.org.
