# Supplementary material for: Emerging Allergens in Goji Berry Superfruit: The Identification of New IgE Binding Proteins towards Allergic Patients’ Sera
Source: Biomolecules. 2020 Apr 29;10(5):689. doi: 10.3390/biom10050689 (PMC7277879; doi:10.3390/biom10050689)
Supplement: Supplementary file 1 [file biomolecules-10-00689-s001.pdf]

Table S1. Full list of the total protein hits retrieved by Proetome discoverer software screening.

N.	Accession	Description	Coverage (%)	Peptides (unique)	PSMs	Score Sequest HT
1	A0A0A7DVY6	Fibrillin OS=Lycium barbarum	69,16	18 (13)	302	524,75
2	A0A3Q7IWI5	Uncharacterized protein OS=Solanum lycopersicum	23,21	11 (4)	187	271,84
3	A0A1J6JXR5	Glutelin type-b 5 OS=Nicotiana attenuata	22,53	12 (4)	196	247,78
4	A0A1U7X9B2	11S globulin seed storage protein 2-like OS=Nicotiana sylvestris	19,02	10 (2)	116	87,44
5	M1B1M5	Uncharacterized protein OS=Solanum tuberosum	15,81	9 (1)	115	81,78
6	A0A3Q7FEY7	Uncharacterized protein OS=Solanum lycopersicum	4,52	8 (1)	81	67,30
7	A0A1J6L9S7	11s globulin subunit beta OS=Nicotiana attenuata	17,10	13 (1)	124	66,05
8	A0A1U7VG63	legumin B-like OS=Nicotiana sylvestris	21,17	12 (2)	119	55,54
9	A0A2G9GMS2	Uncharacterized protein OS=Handroanthus impetiginosus	12,66	4 (1)	41	53,98
10	A0A144YUS5	Ribulose bisphosphate carboxylase large chain OS=Iochroma lehmannii	23,69	10 (2)	75	53,58
11	A0A022S2J1	Uncharacterized protein OS=Erythranthe guttata	5,18	3 (2)	48	53,58
12	G0WZI6	Ribulose bisphosphate carboxylase large chain (Fragment) OS=Solanum chenopodioides	16,56	7 (1)	42	51,71
13	A0A2G2X5V4	11S globulin seed storage protein 2 OS=Capsicum baccatum	14,95	9 (2)	89	50,63

14	A0A1U7W1Q9	vicilin-like antimicrobial peptides 2-3 OS=Nicotiana sylvestris	10,80	9 (6)	92	49,82
15	M1A8H0	Uncharacterized protein OS=Solanum tuberosum	66,67	5 (4)	56	46,28
16	A0A2R2JFS1	SM80.1 Vicilin OS=Solanum melongena	14,79	7 (3)	77	44,34
17	Q948T8	Histone H4 (Fragment) OS=Citrus jambhiri	45,10	5 (2)	40	43,92
18	A0A1U8GRB4	catechol oxidase B, chloroplastic OS=Capsicum annuum	19,35	10 (2)	74	41,66
19	A0A2C9V2W2	Uncharacterized protein OS=Manihot esculenta	22,99	10 (0)	91	38,72
20	A0A103XB50	Histone H4 OS=Cynara cardunculus var. scolymus	45,63	5 (1)	46	36,74
21	M1ANI2	Uncharacterized protein OS=Solanum tuberosum	49,59	6 (3)	37	35,68
22	A0A1U8F773	Uncharacterized protein OS=Capsicum annuum	8,89	4 (1)	47	34,82
23	A0A059AJT0	Uncharacterized protein OS=Eucalyptus grandis	18,40	7 (1)	75	34,60
24	A0A1S4BK33	11S globulin subunit beta-like OS=Nicotiana tabacum	11,58	7 (2)	70	33,12
25	A7UGG9	Non-specific lipid-transfer protein OS=Solanum tuberosum	17,54	2 (1)	21	30,61
26	Q40151	Hsc70 OS=Solanum lycopersicum	18,28	6 (1)	63	27,21
27	B3A0N2	Non-specific lipid-transfer protein (Fragments) OS=Lycium barbarum	39,22	1 (1)	21	26,93
28	F1DBB8	Chloroplast polyphenol oxidase OS=Solanum melongena	20,31	7 (1)	68	26,24
29	C0SQK3	Elongation factor1-alpha (Fragment) OS=Rosa hybrid cultivar	14,63	3 (0)	35	26,01

30	K4D1U9	Non-specific lipid-transfer protein OS=Solanum lycopersicum	25,44	2 (1)	27	25,73
31	O81536	Annexin OS=Solanum lycopersicum	29,94	8 (6)	68	25,62
32	A0A3Q7I4W4	Uncharacterized protein OS=Solanum lycopersicum	10,40	3 (0)	36	24,95
33	O49912	Polyphenol oxidase (Fragment) OS=Nicotiana tabacum	15,54	9 (2)	43	24,12
34	M1A5V5	Uncharacterized protein OS=Solanum tuberosum	29,81	6 (4)	37	23,72
35	A0A3Q7I7H2	Uncharacterized protein OS=Solanum lycopersicum	12,48	6 (1)	53	23,41
36	A0A1S3XNB9	vicilin-like antimicrobial peptides 2-3 OS=Nicotiana tabacum	10,66	4 (1)	43	22,88
37	A0A061ETP3	GTP binding Elongation factor Tu family protein OS=Theobroma cacao	11,84	4 (1)	46	22,77
38	A0A1S4BZG9	Non-specific lipid-transfer protein OS=Nicotiana tabacum	10,95	3 (2)	28	22,04
39	A0A1U7WUU1	light-induced protein, chloroplastic-like OS=Nicotiana sylvestris	18,23	4 (0)	44	20,63
40	A0A3Q7H3K1	Uncharacterized protein OS=Solanum lycopersicum	6,11	5 (1)	55	20,22
41	A0A1U7W0N2	Pectinesterase OS=Nicotiana sylvestris	6,80	4 (1)	44	19,34
42	Q9MVF2	Ribulose bisphosphate carboxylase large chain OS=Nyssa sylvatica	5,68	3 (1)	17	19,08
43	A0A0V0I7Z5	Fructose-bisphosphate aldolase OS=Solanum chacoense	25,00	5 (3)	43	18,79

44	A0A061GW22	HSP20-like chaperones superfamily protein OS=Theobroma cacao	25,79	5 (1)	34	18,43
45	Q41128	Legumin OS=Quercus robur	2,05	1 (1)	11	17,90
46	A0A2U1QHD3	Uncharacterized protein OS=Artemisia annua	6,74	3 (2)	26	17,66
47	A0A023HHL5	Phytoene desaturase OS=Lycium ruthenicum	18,56	3 (2)	34	16,39
48	A0A2P5C6D9	Heat shock protein 70 family OS=Parasponia andersonii	30,10	3 (1)	19	15,86
49	A0A2C9WER1	Uncharacterized protein OS=Manihot esculenta	7,26	3 (1)	16	15,86
50	A0A1U7UPI3	beta-fructofuranosidase, insoluble isoenzyme 1-like isoform X1 OS=Nicotiana sylvestris	9,61	4 (2)	27	14,58
51	A0A1U7X8J8	vicilin-like antimicrobial peptides 2-3 OS=Nicotiana sylvestris	9,98	3 (1)	21	13,68
52	A0A1U7VCA6	17.6 kDa class I heat shock protein-like OS=Nicotiana sylvestris	31,30	3 (0)	24	13,68
53	H6TB43	HSP18.2A OS=Citrullus lanatus	13,13	3 (1)	11	13,68
54	A0A1S3CIN2	18.1 kDa class I heat shock protein-like OS=Cucumis melo	22,01	4 (1)	29	13,68
55	A0A1U8ECH0	Non-specific lipid-transfer protein OS=Capsicum annuum	12,50	2 (1)	22	13,61
56	A0A3Q7HX95	Fructose-bisphosphate aldolase OS=Solanum lycopersicum	17,04	4 (0)	25	12,85
57	A0A3Q7HC76	Uncharacterized protein OS=Solanum lycopersicum	15,78	6 (0)	46	11,82
58	A0A2G2XSR6	Uncharacterized protein OS=Capsicum baccatum	17,17	4 (4)	20	11,29

59	A0A3P6A3B9	Uncharacterized protein OS=Brassica campestris	5,65	3 (2)	17	10,65
60	A0A1S3Y298	Oleosin OS=Nicotiana tabacum	13,43	2 (1)	21	10,52
61	A0A1Q3BEJ8	Cupin_1 domain-containing protein (Fragment) OS=Cephalotus follicularis	6,52	3 (2)	17	9,97
62	A0A328D894	Uncharacterized protein OS=Cuscuta australis	22,55	3 (0)	34	9,31
63	M1CHX3	Uncharacterized protein OS=Solanum tuberosum	14,89	2 (1)	19	9,30
64	A0A3P6BEI0	Uncharacterized protein OS=Brassica oleracea	11,83	5 (0)	38	9,26
65	A0A2G2VZT2	Non-specific lipid-transfer protein OS=Capsicum baccatum	9,52	1 (1)	8	8,84
66	Q8GZP6	Allergen Ana o 2 (Fragment) OS=Anacardium occidentale	5,47	3 (2)	12	8,58
67	A0A314L9V3	Sucrose-binding protein OS=Nicotiana attenuata	11,09	3 (0)	26	8,22
68	A0A1R3IHM8	Fructose-bisphosphate aldolase OS=Corchorus capsularis	13,69	3 (0)	21	7,71
69	A0A1S3Z0N4	Fructose-bisphosphate aldolase OS=Nicotiana tabacum	15,08	4 (0)	26	7,71
70	A0A059A4U5	Fructose-bisphosphate aldolase OS=Eucalyptus grandis	14,25	3 (1)	19	7,71
71	A0A1U8F3Z9	V-type proton ATPase catalytic subunit A-like OS=Capsicum annuum	14,29	4 (4)	33	7,24
72	A0A2S1TKL0	Ribulose bisphosphate carboxylase large chain OS=Ipomoea hederacea var. integriuscula	13,54	6 (0)	29	7,11
73	A0A2N9FMY0	ATP synthase subunit beta OS=Fagus sylvatica	21,82	5 (0)	38	6,88

74	A0A328D7A1	ATP synthase subunit beta OS=Cuscuta australis	22,76	5 (0)	38	6,88
75	A0A1J6IW81	Chaperonin 60 subunit beta 2, chloroplastic OS=Nicotiana attenuata	16,03	3 (2)	29	6,73
76	A0A0V0IB00	Putative enolase-like OS=Solanum chacoense	16,44	4 (2)	17	6,32
77	A0A164WJJ6	Uncharacterized protein OS=Daucus carota subsp. sativus	10,39	3 (0)	17	6,05
78	A0A061F0S7	Enolase OS=Theobroma cacao	11,69	3 (0)	16	6,05
79	A0A2H5PF70	Uncharacterized protein OS=Citrus unshiu	35,07	5 (1)	33	5,99
80	A0A1J3JA24	Mediator of RNA polymerase II transcription subunit 37f OS=Noccaea caerulescens	9,57	4 (2)	20	5,95
81	A0A3P5ZBN3	Pectinesterase OS=Brassica campestris	5,57	4 (0)	32	5,88
82	B9ZUJ0	EF-1-alpha (Fragment) OS=Olea europaea	26,67	3 (1)	24	5,83
83	A0A2P5BGR5	Fructose-bisphosphate aldolase OS=Parasponia andersonii	20,35	4 (2)	36	5,56
84	A0A3Q7GMW1	Uncharacterized protein OS=Solanum lycopersicum	4,46	4 (3)	15	5,43
85	A0A410SNJ0	Ribulose-1,5-bisphosphate carboxylase/oxygenase large subunit OS=Lycium barbarum	7,64	3 (1)	20	5,27
86	A0A2Z6MJE1	Kinesin-like protein OS=Trifolium subterraneum	2,99	3 (2)	19	5,24
87	W9SCU3	Fructose-bisphosphate aldolase OS=Morus notabilis	13,73	4 (1)	18	5,22
88	A0A2K3NGD9	ATP synthase subunit beta (Fragment) OS=Trifolium pratense	14,40	5 (1)	28	5,13

89	A0A1J7HJE4	ATP synthase subunit beta OS=Lupinus angustifolius	15,71	5 (0)	31	5,13
90	A0A1S4BAC6	aspartyl protease AED3-like OS=Nicotiana tabacum	14,84	3 (1)	23	5,07
91	A0A328CYB7	Histone H2B OS=Cuscuta australis	32,45	5 (3)	42	5,02
92	A0A061FP02	Uncharacterized protein OS=Theobroma cacao	7,63	1 (1)	8	4,98
93	A0A397ZLP9	Uncharacterized protein OS=Brassica campestris	14,29	1 (1)	4	4,97
94	Q8L5C8	Malate dehydrogenase OS=Solanum tuberosum	23,26	2 (1)	20	4,16
95	A0A2N9GJV1	Uncharacterized protein OS=Fagus sylvatica	2,02	3 (1)	10	4,03
96	V4UHK2	Uncharacterized protein OS=Citrus clementina	3,39	3 (2)	19	4,03
97	I3SR52	Pectinesterase OS=Lotus japonicus	9,23	4 (1)	13	4,01
98	A0A061GWL6	HSP20-like chaperones superfamily protein OS=Theobroma cacao	14,56	3 (1)	21	3,83
99	A0A2G3C470	Cytochrome 97B3, chloroplastic OS=Capsicum chinense	6,94	3 (3)	25	3,66
100	M4CYR6	ATP synthase subunit beta OS=Brassica rapa subsp. pekinensis	13,51	4 (1)	33	3,45
101	Q6WB92	Enolase OS=Gossypium barbadense	8,09	3 (1)	10	3,44
102	A0A1R3IXS3	Uncharacterized protein OS=Corchorus capsularis	5,87	4 (2)	20	3,38
103	A0A251PKB1	Uncharacterized protein OS=Prunus persica	6,76	3 (2)	17	3,27
104	A0A2J6MDX7	Uncharacterized protein OS=Lactuca sativa	6,10	1 (1)	6	3,13
105	A0A218VX25	Uncharacterized protein OS=Punica granatum	4,59	3 (0)	23	2,80

106	A0A103YHD2	AAA+ ATPase domain-containing protein OS=Cynara cardunculus var. scolymus	8,45	4 (1)	23	2,80
107	A0A2C9W2S4	Pectinesterase OS=Manihot esculenta	5,68	5 (1)	17	2,02
108	A0A2R6PB96	Acyl-acyl carrier protein like OS=Actinidia chinensis var. chinensis	14,35	3 (2)	7	1,95
109	A0A2C9UA00	Pectinesterase OS=Manihot esculenta	3,07	3 (1)	10	1,95
110	F1DBB7	Chloroplast polyphenol oxidase (Fragment) OS=Solanum melongena	17,98	4 (0)	22	1,80
111	A0A1S3TTZ7	beta-glucosidase 11 OS=Vigna radiata var. radiata	4,67	3 (1)	15	1,71
112	A0A1J6L287	Glycine-rich rna-binding protein OS=Nicotiana attenuata	20,51	3 (2)	12	1,68
113	A0A3Q7GS13	Pectinesterase OS=Solanum lycopersicum	6,03	3 (1)	20	1,41
114	A0A2K3LAT7	Heat shock cognate 70 kDa protein 2-like (Fragment) OS=Trifolium pratense	2,13	3 (0)	11	1,38
115	G8E552	Ribulose-1,5-bisphophate carboxylase oxygenase (Fragment) OS=Aglaia sp. PA3E0160	7,20	2 (1)	3	1,37

Table S2. Cross-reactivity of the protein identified in the total GB digest by proteomic discovery MS analysis and by searching and aligning the full length sequences on allergenoline.

N.	Accession	Description	Accession (uniprot)	Description	E-values	% Identity	Allergen code (Allergome)
1	A0A0A7DVY6	Fibrillin OS=Lycium barbarum	/	/	/	/	/
**2**	**A0A3Q7IWI5**	**Uncharacterized protein OS=Solanum lycopersicum**	**Q84ND2**	**11S globulin [Bertholletia excelsa]**	**9.6e-114**	**55.0%**	**Ber e 2.0101/Ber e 2**
**3**	**A0A1J6JXR5**	**Glutelin type-b 5 OS=Nicotiana attenuata**	**A0EM47**	**11S globulin-like protein [Actinidia chinensis]**	**1.1e-069**	**57.4%**	**Act d 12.0102**
4	A0A1U7X9B2	11S globulin seed storage protein 2-like OS=Nicotiana sylvestris	/	/	/	/	/
**5**	**M1B1M5**	**Uncharacterized protein OS=Solanum tuberosum**	**B7P073**	**11S globulin precusor [Pistacia vera]**	**5.5e-084**	**50.0%**	**Pis v 2/Pis v 2.0101**
**6**	**A0A3Q7FEY7**	**Uncharacterized protein OS=Solanum lycopersicum**	**Q9AUD2**	**11S globulin [Sesamum indicum]**	**4.4e-046**	**54.8%**	**Ses i 7/Ses i 7.0101**
**7**	**A0A1J6L9S7**	**11s globulin subunit beta OS=Nicotiana attenuata**	**B7P073**	**11S globulin precusor [Pistacia vera]**	**3.1e-109**	**53.3%**	**Pis v 2/Pis v 2.0101**
**8**	**A0A1U7VG63**	**legumin B-like OS=Nicotiana sylvestris**	**B7P074**	**11S globulin precusor [Pistacia vera]**	**4.1e-105**	**52.0%**	**Pis v 2/Pis v 2.0102**
9	A0A2G9GMS2	Uncharacterized protein OS=Handroanthus impetiginosus	/	/	/	/	/
10	A0A144YUS5	Ribulose bisphosphate carboxylase large chain OS=Iochroma lehmannii	/	/	/	/	/
**11**	**A0A022S2J1**	**Uncharacterized protein OS=Erythranthe guttata**	**Q9XHP0**	**11S globulin precursor (Sesamum indicum)**	**9.1e-154**	**72.8%**	**Ses i 6**
12	G0WZI6	Ribulose bisphosphate carboxylase large chain (Fragment) OS=Solanum chenopodioides	/	/	/	/	/
**13**	**A0A2G2X5V4**	**11S globulin seed storage protein 2 OS=Capsicum baccatum**	**Q9XHP0**	**11S globulin precursor [Sesamum indicum]**	**5,00E-102**	**62.1%**	**Ses i 6**
14	A0A1U7W1Q9	vicilin-like antimicrobial peptides 2-3 OS=Nicotiana sylvestris	/	/	/	/	/
15	M1A8H0	Uncharacterized protein OS=Solanum tuberosum	/	/	/	/	/

16	A0A2R2JFS1	SM80.1 Vicilin OS=Solanum melongena	/	/	/	/	/
17	Q948T8	Histone H4 (Fragment) OS=Citrus jambhiri	/	/	/	/	/
18	A0A1U8GRB4	catechol oxidase B, chloroplastic OS=Capsicum annuum	/	/	/	/	/
**19**	**A0A2C9V2W2**	**Uncharacterized protein OS=Manihot esculenta**	**A0A1B2YLJ2**	**Heat shock-like protein (Tyrophagus putrescentia)**	**3.4e-211**	**73.9%**	**Tyr p 28/Tyr p 28.0101**
			**P40918**	**Heat shock 70 kDa protein (Davidiella tassiana)**	**1.9e-200**	**71.7%**	**Cla h HSP70**
			**A0A088SAS1**	**Der f 28 allergen (Dermatophagoides farinae)**	**2.1e-199**	**70.4%**	**Der f 28/Der f 28.0201**
20	A0A103XB50	Histone H4 OS=Cynara cardunculus var. scolymus	/	/	/	/	/
**21**	**M1ANI2**	**Uncharacterized protein OS=Solanum tuberosum**	**Q9XHP0**	**11S globulin precursor [Sesamum indicum]**	**4.7e-031**	**66.7%**	**Ses i 6**
22	A0A1U8F773	Uncharacterized protein OS=Capsicum annuum	/	/	/	/	/
**23**	**A0A059AJT0**	**Uncharacterized protein OS=Eucalyptus grandis**	**A0A1B2YLJ2**	**Heat shock-like protein (Tyrophagus putrescentia)**	**4.5e-168**	**73.9%**	**Tyr p 28/Tyr p 28.0101**
			**P40918**	**Heat shock 70 kDa protein (Davidiella tassiana)**	**2.2e-161**	**72.0%**	**Cla h HSP70**
			**A0A088SAS1**	**Der f 28 allergen (Dermatophagoides farinae)**	**5.5e-160**	**70.6%**	**Der f 28/Der f 28.0201**
24	A0A1S4BK33	11S globulin subunit beta-like OS=Nicotiana tabacum	/	/	/	/	/
**25**	**A7UGG9**	**Non-specific lipid-transfer protein OS=Solanum tuberosum**	**P93224**	**non specific lipid transfer protein (Solanum lycopersicum)**	**3.7e-039**	**93.9%**	**Sola l 3/Sola l 3.0101**
			**Q4A1N1**	**non-specific lipid transfer protein (Lycopersicon)**	**3.9e-031**	**78.1%**	**Sola l 3**
**26**	**Q40151**	**Hsc70 OS=Solanum lycopersicum**	**A0A1B2YLJ2**	**Heat shock-like protein (Tyrophagus putrescentia)**	**2.1e-209**	**74.1%**	**Tyr p 28/Tyr p 28.0101**
			**P40918**	**Heat shock 70 kDa protein (Davidiella tassiana)**	**4.6e-199**	**71.0%**	**Cla h HSP70**
			**A0A088SAS1**	**Der f 28 allergen (Dermatophagoides farinae)**	**8.3e-200**	**70.5%**	**Der f 28/Der f 28.0201**
**27**	**B3A0N2**	**Non-specific lipid-transfer protein (Fragments) OS=Lycium barbarum**	**B3A0N2**	**Non-specific lipid-transfer protein (Lycium barbarum)**	**1.6e-016**	**100.0%**	**Lyc ba 3**

			**W0U0V5**	**Non-specific lipid-transfer protein (Cannabis sativa)**	**4.3e-008**	**72.5%**	**Can s 3.0101/Can s 3**
			**A0A059STC4**	**Non specific lipid transfer protein 1A (Punica granatum)**	**4.3e-008**	**75.0%**	**Pun g 1.0101/Pun g 1**
28	F1DBB8	Chloroplast polyphenol oxidase OS=Solanum melongena	/	/	/	/	/
29	C0SQK3	Elongation factor1-alpha (Fragment) OS=Rosa hybrid cultivar	/	/	/	/	/
**30**	**K4D1U9**	**Non-specific lipid-transfer protein OS=Solanum lycopersicum**	**P93224**	**Non specific lipid transfer protein [Solanum lycopersicum]**	**2.9e-036**	**79.8%**	**Sola l 3/Sola l 3.0101**
			**Q4A1N1**	**Non-specific lipid transfer protein [Solanum lycopersicum]**	**3.4e-032**	**72.8%**	**Sola l 3**
31	O81536	Annexin OS=Solanum lycopersicum	/	/	/	/	/
32	A0A3Q7I4W4	Uncharacterized protein OS=Solanum lycopersicum	/	/	/	/	/
33	O49912	Polyphenol oxidase (Fragment) OS=Nicotiana tabacum	/	/	/	/	/
34	M1A5V5	Uncharacterized protein OS=Solanum tuberosum	/	/	/	/	/
35	A0A3Q7I7H2	Uncharacterized protein OS=Solanum lycopersicum	/	/	/	/	/
**36**	**A0A1S3XNB9**	**vicilin-like antimicrobial peptides 2-3 OS=Nicotiana tabacum**	**Q9AUD0**	**7S globulin (Sesamum indicum)**	**2.3e-135**	**66.6%**	**Ses i 3.0101/Ses i 3**
37	A0A061ETP3	GTP binding Elongation factor Tu family protein OS=Theobroma cacao	/	/	/	/	/
38	A0A1S4BZG9	Non-specific lipid-transfer protein OS=Nicotiana tabacum	/	/	/	/	/
39	A0A1U7WUU1	light-induced protein, chloroplastic-like OS=Nicotiana sylvestris	/	/	/	/	/
**40**	**A0A3Q7H3K1**	**Uncharacterized protein OS=Solanum lycopersicum**	**A0A1B2YLJ2**	**Heat shock-like protein (Tyrophagus putrescentia)**	**1.9e-157**	**71.7%**	**Tyr p 28/Tyr p 28.0101**
			**P40918**	**Heat shock 70 kDa protein (Davidiella tassiana)**	**2.1e-150**	**69.2%**	**Cla h HSP70**
			**A0A088SAS1**	**Der f 28 allergen (Dermatophagoides farinae)**	**7.3e-150**	**68.3%**	**Der f 28/Der f 28.0201**
41	A0A1U7W0N2	Pectinesterase OS=Nicotiana sylvestris	/	/	/	/	/

42	Q9MVF2	Ribulose bisphosphate carboxylase large chain OS=Nyssa sylvatica	/	/	/	/	/
43	A0A0V0I7Z5	Fructose-bisphosphate aldolase OS=Solanum chacoense	/	/	/	/	/
44	A0A061GW22	HSP20-like chaperones superfamily protein OS=Theobroma cacao	/	/	/	/	/
**45**	**Q41128**	**Legumin OS=Quercus robur**	**B5KVH4**	**11S legumin protein (Carya illinoinensis)**	**3,00E-65**	**66.5%**	**Car i 4/Car i 4.0101**
46	A0A2U1QHD3	Uncharacterized protein OS=Artemisia annua	/	/	/	/	/
47	A0A023HHL5	Phytoene desaturase OS=Lycium ruthenicum	/	/	/	/	/
**48**	**A0A2P5C6D9**	**Heat shock protein 70 family OS=Parasponia andersonii**	**A0A1B2YLJ2**	**Heat shock-like protein [Tyrophagus putrescentiae]**	**6.4e-036**	**83.8%**	**Tyr p 28/Tyr p 28.0101**
			**P40918**	**Heat shock 70 kDa protein (Davidiella tassiana)**	**3.2e-033**	**75.7%**	**Cla h HSP70**
			**A0A088SAS1**	**Der f 28 allergen [Dermatophagoides farinae]**	**2,00E-33**	**78.4%**	**Der f 28/Der f 28.0201**
			**L7V065**	**Heat shock protein 70 [Dermatophagoides farinae]**	**4.7e-035**	**81.1%**	**Der f 28/Der f 28.0201**
**49**	**A0A2C9WER1**	**Uncharacterized protein OS=Manihot esculenta**	**A0A1B2YLJ2**	**Heat shock-like protein [Tyrophagus putrescentiae]**	**1,00E-210**	**74.1%**	**Tyr p 28/Tyr p 28.0101**
			**P40918**	**Heat shock 70 kDa protein (Davidiella tassiana)**	**7.4e-199**	**71.4%**	**Cla h HSP70**
			**A0A088SAS1**	**Der f 28 allergen [Dermatophagoides farinae]**	**5.8e-198**	**70.4%**	**Der f 28/Der f 28.0201**
50	A0A1U7UPI3	beta-fructofuranosidase, insoluble isoenzyme 1-like isoform X1 OS=Nicotiana sylvestris	/	/	/	/	/
51	A0A1U7X8J8	vicilin-like antimicrobial peptides 2-3 OS=Nicotiana sylvestris	/	/	/	/	/
52	A0A1U7VCA6	17.6 kDa class I heat shock protein-like OS=Nicotiana sylvestris	/	/	/	/	/
53	H6TB43	HSP18.2A OS=Citrullus lanatus	/	/	/	/	/
54	A0A1S3CIN2	18.1 kDa class I heat shock protein-like OS=Cucumis melo	/	/	/	/	/
55	A0A1U8ECH0	Non-specific lipid-transfer protein OS=Capsicum annuum					

**56**	**A0A3Q7HX95**	**Fructose-bisphosphate aldolase OS=Solanum lycopersicum**	**P86979**	**Fructose-bisphosphate aldolase A (Thunnus albacares)**	**0.0028**	**70.8%**	**Thu a 3/Thu a 3.0101**
57	A0A3Q7HC76	Uncharacterized protein OS=Solanum lycopersicum	/	/	/	/	/
58	A0A2G2XSR6	Uncharacterized protein OS=Capsicum baccatum	/	/	/	/	/
59	A0A3P6A3B9	Uncharacterized protein OS=Brassica campestris	/	/	/	/	/
**60**	**A0A1S3Y298**	**Oleosin OS=Nicotiana tabacum**	**B5TMA5**	**15 kDa oleosin [Sesamum indicum]**	**3.5e-032**	**59.8%**	**Ses i 5.0101**
**61**	**A0A1Q3BEJ8**	**Cupin_1 domain-containing protein (Fragment) OS=Cephalotus follicularis**	**B5KVH5**	**11S legumin protein [Carya illinoinensis]**	**8.9e-055**	**57.2%**	**Car i 4.0101**
62	A0A328D894	Uncharacterized protein OS=Cuscuta australis	/	/	/	/	/
**63**	**M1CHX3**	**Uncharacterized protein OS=Solanum tuberosum**	**NP_001306883 (NCBI)**	**Lipid-transfer protein 7k-LTP precursor (Solanum lycopersicum)**	**5.6e-021**	**93.6%**	**Sola l 6.0101**
64	A0A3P6BEI0	Uncharacterized protein OS=Brassica oleracea	/	/	/	/	/
65	A0A2G2VZT2	Non-specific lipid-transfer protein OS=Capsicum baccatum	/	/	/	/	/
**66**	**Q8GZP6**	**Allergen Ana o 2 (Fragment) OS=Anacardium occidentale**	**Q8GZP6**	**11S globulin seed storage protein Ana o 2.0101 (Anacardium occidentale)**	**3.1e-138**	**100%**	**Ana o 2.0101/Ana o 2**
**67**	**A0A314L9V3**	**Sucrose-binding protein OS=Nicotiana attenuata**	**Q9AUD0**	**7S globulin [Sesamum indicum]**	**2.1e-134**	**66.5%**	**Ses i 3.0101, Ses i 3**
**68**	**A0A1R3IHM8**	**Fructose-bisphosphate aldolase OS=Corchorus capsularis**	**B5DGM7**	**aldolase a, fructose-bisphosphate 1 [Salmo salar]**	**4.5e-107**	**61.9%**	**Sal s 3/ Sal s 3.0101**
**69**	**A0A1S3Z0N4**	**Fructose-bisphosphate aldolase OS=Nicotiana tabacum**	**D4HTS6**	**aldolase A [Thunnus albacares]**	**5.5e-115**	**60.6%**	**Thu a 3**
**70**	**A0A059A4U5**	**Fructose-bisphosphate aldolase OS=Eucalyptus grandis**	**B5DGM7**	**aldolase a, fructose-bisphosphate 1 [Salmo salar]**	**1.3e-090**	**60.8%**	**Sal s 3/ Sal s 3.0101**
71	A0A1U8F3Z9	V-type proton ATPase catalytic subunit A-like OS=Capsicum annuum	/	/	/	/	/
72	A0A2S1TKL0	Ribulose bisphosphate carboxylase large chain OS=Ipomoea hederacea var. integriuscula	/	/	/	/	/
73	A0A2N9FMY0	ATP synthase subunit beta OS=Fagus sylvatica	/	/	/	/	/

74	A0A328D7A1	ATP synthase subunit beta OS=Cuscuta australis	/	/	/	/	/
75	A0A1J6IW81	Chaperonin 60 subunit beta 2, chloroplastic OS=Nicotiana attenuata	/	/	/	/	/
**76**	**A0A0V0IB00**	**Putative enolase-like OS=Solanum chacoense**	**Q9LEJ0**	**Enolase 1 (Hevea brasiliensis)**	**4,00E-172**	**88,7%**	**Hev b 9.0101/Hev b 9**
			**Q9LEI9**	**Enolase 2 (Hevea brasiliensis)**	**1,00E-171**	**88,5%**	**Hev b 9.0101/Hev b 9**
			**I0J1J2**	**Enolase (Salmo salar)**	**1.2e-131**	**71,0%**	**Sal s 2**
			**I0J1J1**	**Alpha-enolase (Thunnus albacares)**	**9.8e-130**	**69,5%**	**Thu a 2**
			**B5DGQ7**	**Beta-enolase (Salmo salar)**	**4.4e-128**	**69,6%**	**Sal s 2/Sal s 2.0101**
**77**	**A0A164WJJ6**	**Uncharacterized protein OS=Daucus carota subsp. sativus**	**Q9LEI9**	**Enolase 2 (Hevea brasiliensis)**	**1.1e-095**	**81,0%**	**Hev b 9.0101/Hev b 9**
			**Q9LEJ0**	**Enolase 1 (Hevea brasiliensis)**	**4.4e-095**	**80,8%**	**Hev b 9.0101/Hev b 9**
**78**	**A0A061F0S7**	**Enolase OS=Theobroma cacao**	**Q9LEI9**	**Enolase 2 (Hevea brasiliensis)**	**2.4e-177**	**91,2%**	**Hev b 9.0101/Hev b 9**
			**Q9LEJ0**	**Enolase 1 (Hevea brasiliensis)**	**1.6e-176**	**90,8%**	**Hev b 9.0101/Hev b 9**
			**I0J1J2**	**Enolase (Salmo salar)**	**3.7e-128**	**69,3%**	**Sal s 2**
**79**	**A0A2H5PF70**	**Uncharacterized protein OS=Citrus unshiu**	**C7C4X1**	**Glyceraldehyde-3-phosphate dehydrogenase (Triticum aestivum)**	**3.1e-062**	**75,1%**	**Tri a 34/Tri a 34.0101**
**80**	**A0A1J3JA24**	**Mediator of RNA polymerase II transcription subunit 37f OS=Noccaea caerulescens**	**Q1HR69**	**Heat shock cognate 70 [Aedes aegypti]**	**4.7e-197**	**68.7%**	**Aed a 8.0101/Aed a 8**
81	A0A3P5ZBN3	Pectinesterase OS=Brassica campestris	/	/	/	/	/
82	B9ZUJ0	EF-1-alpha (Fragment) OS=Olea europaea	/	/	/	/	/
**83**	**A0A2P5BGR5**	**Fructose-bisphosphate aldolase OS=Parasponia andersonii**	**A0A068FCL9**	**Pen c 1 allergen [Penaeus chinensis]**	**1.5e-071**	**52.6%**	**Unassigned**
84	A0A3Q7GMW1	Uncharacterized protein OS=Solanum lycopersicum	/	/	/	/	/
85	A0A410SNJ0	Ribulose-1,5-bisphosphate carboxylase/oxygenase large subunit OS=Lycium barbarum	/	/	/	/	/

86	A0A2Z6MJE1	Kinesin-like protein OS=Trifolium subterraneum	/	/	/	/	/
**87**	**W9SCU3**	**Fructose-bisphosphate aldolase OS=Morus notabilis**	**A0A068FCL9**	**Pen c 1 allergen [Penaeus chinensis]**	**1.3e-062**	**50.1%**	**Unassigned**
88	A0A2K3NGD9	ATP synthase subunit beta (Fragment) OS=Trifolium pratense	/	/	/	/	/
89	A0A1J7HJE4	ATP synthase subunit beta OS=Lupinus angustifolius	/	/	/	/	/
90	A0A1S4BAC6	aspartyl protease AED3-like OS=Nicotiana tabacum	/	/	/	/	/
91	A0A328CYB7	Histone H2B OS=Cuscuta australis	/	/	/	/	/
92	A0A061FP02	Uncharacterized protein OS=Theobroma cacao	/	/	/	/	/
93	A0A397ZLP9	Uncharacterized protein OS=Brassica campestris	/	/	/	/	/
**94**	**Q8L5C8**	**Malate dehydrogenase OS=Solanum tuberosum**	**Q9Y750**	**major allergenic protein Mal f4 [Malassezia furfur]**	**2,00E-65**	**52.5%**	**Mala f 4.0101/Mala f 4**
95	A0A2N9GJV1	Uncharacterized protein OS=Fagus sylvatica	/	/	/	/	/
96	V4UHK2	Uncharacterized protein OS=Citrus clementina	/	/	/	/	/
97	I3SR52	Pectinesterase OS=Lotus japonicus	/	/	/	/	/
98	A0A061GWL6	HSP20-like chaperones superfamily protein OS=Theobroma cacao	/	/	/	/	/
99	A0A2G3C470	Cytochrome 97B3, chloroplastic OS=Capsicum chinense	/	/	/	/	/
100	M4CYR6	ATP synthase subunit beta OS=Brassica rapa subsp. pekinensis	/	/	/	/	/
**101**	**Q6WB92**	**Enolase OS=Gossypium barbadense**	**Q9LEI9**	**Enolase 2 (Hevea brasiliensis)**	**2.6e-193**	**90,80%**	**Hev b 9.0101/Hev b 9**
			**Q9LEJ0**	**Enolase 1 (Hevea brasiliensis)**	**3,00E-193**	**90,80%**	**Hev b 9.0101/Hev b 9**
			**I0J1J2**	**Enolase (Salmo salar)**	**3.9e-142**	**69,90%**	**Sal s 2**
			**I0J1J1**	**Alpha-enolase (Thunnus albacares)**	**1.8e-141**	**69,50%**	**Thu a 2**
102	A0A1R3IXS3	Uncharacterized protein OS=Corchorus capsularis	/	/	/	/	/

103	A0A251PKB1	Uncharacterized protein OS=Prunus persica	/	/	/	/	/
104	A0A2J6MDX7	Uncharacterized protein OS=Lactuca sativa	/	/	/	/	/
105	A0A218VX25	Uncharacterized protein OS=Punica granatum	/	/	/	/	/
106	A0A103YHD2	AAA+ ATPase domain-containing protein OS=Cynara cardunculus var. scolymus	/	/	/	/	/
107	A0A2C9W2S4	Pectinesterase OS=Manihot esculenta	/	/	/	/	/
108	A0A2R6PB96	Acyl-acyl carrier protein like OS=Actinidia chinensis var. chinensis	/	/	/	/	/
109	A0A2C9UA00	Pectinesterase OS=Manihot esculenta	/	/	/	/	/
110	F1DBB7	Chloroplast polyphenol oxidase (Fragment) OS=Solanum melongena	/	/	/	/	/
111	A0A1S3TTZ7	beta-glucosidase 11 OS=Vigna radiata var. radiata	/	/	/	/	/
112	A0A1J6L287	Glycine-rich rna-binding protein OS=Nicotiana attenuata	/	/	/	/	/
113	A0A3Q7GS13	Pectinesterase OS=Solanum lycopersicum	/	/	/	/	/
**114**	**A0A2K3LAT7**	**Heat shock cognate 70 kDa protein 2-like (Fragment) OS=Trifolium pratense**	**A0A1B2YLJ2**	**heat shock-like protein (Tyrophagus putrescentia)**	**3,00E-139**	**64.8%**	**Tyr p 28/Tyr p 28.0101**
115	G8E552	Ribulose-1,5-bisphophate carboxylase oxygenase (Fragment) OS=Aglaia sp. PA3E0160	/	/	/	/	/
	**A0A1S4CJA8**	**Vicilin like, OS=Nicotiana tabacum (banda a)**	**Q7Y1C1**	**vicilin seed storage protein [Juglans nigra]**	**5.2e-104**	**49.9%**	**Jug n 2.0101/Jug n 2**
	**A0A2G2ZXN5**	**Legumin –like, OS=Capsicum annuum (banda l)**	**Q84ND2**	**11S globulin [Bertholletia excelsa]**	**1.5e-111**	**53.4%**	**Ber e 2.0101/Ber e 2**
